# Kappa index: real-world study for assessing cut-off values that provide the best accuracy for multiple sclerosis diagnosis in patients with CIS

**DOI:** 10.3389/fimmu.2026.1771566

**Published:** 2026-08-25

**Authors:** José Luis Veiga-González, Enric Monreal, Mercedes Espiño, Juan Luis Chico-García, José Ignacio Fernández-Velasco, Fernando Rodríguez-Jorge, Noelia Villarrubia, Alexander Rodero-Romero, Susana Sainz de la Maza, Jaime Masjuan, Lucienne Costa-Frossard, Luisa María Villar

**Affiliations:** 1 Department of Immunology, Hospital Universitario Ramón y Cajal, Red Española de Esclerosis Múltiple (REEM), Red de Enfermedades Inflamatorias (REI), Instituto de Salud Carlos III (ISCIII), Instituto Ramón y Cajal de Investigación Sanitaria (IRYCIS), Madrid, Spain; 2 Department of Neurology, Hospital Universitario Ramón y Cajal, Red Española de Esclerosis Múltiple (RE-EM), Red de Enfermedades Inflamatorias (REI), ISCIII, IRYCIS, Madrid, Spain

**Keywords:** clinically isolated syndrome, diagnosis, kappa free light chain, multiple sclerosis, oligoclonal IgG bands

## Abstract

**Background:**

Intrathecal immunoglobulin G synthesis (ITGS) is important for MS diagnosis. Oligoclonal bands (OCB) have demonstrated sensitivity and specificity, but their complexity necessitates their analysis in specialized laboratories. Measuring the kappa free light chain index emerged as an automated and reproducible alternative and was incorporated as an alternative to OCB in the 2024 McDonald criteria. However, establishing the optimal cut-off for predicting MS diagnosis in patients with clinically isolated syndrome (CIS) is needed.

**Methods:**

We included 391 patients with CIS recruited between 2017 and 2022 who were followed for at least three years. Cerebrospinal fluid and serum samples were analyzed for OCB and the kappa index. Sensitivity, specificity, positive and negative predictive values were calculated for multiple kappa index cut-offs (6.1–20) and compared with OCB, the IgG index, and Reiber formulae. Subgroup analyses were performed according to CIS topography.

**Results:**

A cut-off value of 15, with OCB assessment in cases with lower values, gave the best results in the total cohort, with sensitivity and specificity higher than 90%. However, subgroup analysis revealed two patterns depending on CIS location. In all locations except the optic nerve, Reiber formulae gave excellent results, comparable to those of OCB. Conversely, in patients with optic neuritis, Reiber formulae and the kappa index gave low specificity. The best strategy for these patients was a kappa index cut-off value of 15, with OCB assessment for lower values, or OCB alone.

**Conclusion:**

The optimal semiquantitative method for detecting kappa intrathecal synthesis for predicting MS in patients with CIS depends on CIS topography. A positive value for Reiber formulae or a cut-off of 6.1 provides diagnostic performance comparable to OCB in non-optic neuritis CIS, reducing the need for OCB analysis. In optic neuritis, OCB assessment alone or in conjunction with a kappa index cut-off of 15 offered the best diagnostic accuracy.

## 1 Introduction

An early and trustworthy diagnosis, followed by appropriate treatment, is crucial to avoid disability progression in multiple sclerosis (MS) (1). Different revisions of diagnostic criteria include clinical, magnetic resonance imaging (MRI), and cerebrospinal fluid (CSF) data to contribute to an early MS diagnosis (2, 3). Intrathecal IgG synthesis (ITGS), when explored by oligoclonal bands (OCB) analysis, has a sensitivity ranging from 60% to 100%, but it is approximately 90% when explored by isoelectric focusing and Western blotting (4). It contributes to demonstrating the inflammatory nature of the disease (5, 6). In the 2017 version of the McDonald criteria, it could substitute for dissemination in time, and thus contributed to accelerating MS diagnosis in patients with clinically isolated syndrome (CIS). However, its analysis can be challenging due to the complexity of performing and interpreting the isoelectric focusing technique (7). To facilitate the detection of ITGS, semiquantitative tests, such as Reiber or IgG indexes, were proposed but showed lower sensitivity than OCB (8–10). In recent years, first, kappa free light chain (KFLC) in CSF values and its Reiber diagram and, subsequently, the kappa index have emerged as good candidates for demonstrating ITGS (11–15), with good results in terms of sensitivity, although their specificity appears to be lower (16–18). This lack of specificity may be because the increase in the concentration of kappa light chains in the fluid is not unique to MS but also occurs in other inflammatory diseases of the central nervous system (19).

However, this technique has remarkable advantages. Its automation allows early analysis in non-specialized laboratories, enabling more tests to be performed per day than when OCB are used and providing reproducible results (20, 21). In view of these findings, the 2024 McDonald criteria considered an elevated kappa index as a diagnostic criterion alongside OCB for detecting intrathecal immunoglobulin synthesis (22).

However, some issues should be addressed. The most important is to define the best cut-off for this technique for predicting MS diagnosis in patients with CIS in the clinical setting (17).

We aimed to evaluate the sensitivity, specificity, positive predictive value, and negative predictive value of different cut-off values of the kappa index in a real-life cohort of CIS patients. We also evaluated the value of a putative combination with OCB.

## 2 Methods

### 2.1 Study design

This was a prospective study of patients with CIS recruited at Ramón y Cajal University Hospital and followed prospectively. The study was approved by the Ethics Committee of Ramón y Cajal University Hospital. All patients signed informed consent before entry.

#### 2.1.1 Patients

The inclusion criteria included: having a first demyelinating event with CSF and serum samples available; being prospectively followed in our MS unit until a definite MS diagnosis or for at least three years; having an MRI study performed within six months of symptom onset; being between 18 and 65 years of age; not receiving any disease-modifying therapy (DMT) prior to lumbar puncture (LP); and not having received high-dose steroids in the two months before LP. No patient was on DMT at the time of sample collection, and all had been free of high-dose corticosteroids for at least three months before LP. This strict selection was applied to avoid the known effects of highly effective DMTs and B-cell–depleting therapies on KFLC concentrations, as previously reported (23–25).

The classification of the CIS type throughout the manuscript is based primarily on clinical presentation. In all cases, the diagnosis was independently confirmed by more than two qualified neurologists. In the case of optic neuritis, ophthalmologists participated in the diagnosis. Notably, the majority of optic neuritis cases were initially identified within the ophthalmology department and subsequently referred to Neurology for further evaluation. Diagnostic confirmation was consistently supported by pathological findings in at least one of the three diagnostic tests performed (visual evoked potential, optical coherence tomography, and MRI).

Patients studied in our MS unit from 2017 to 2022 who fulfilled the inclusion criteria and signed informed consent to participate in the study were included consecutively.

### 2.2 Data collection

Experienced neurologists of the MS unit collected demographic, clinical, radiological, and laboratory data at patient inclusion, and follow-up data, including final diagnosis, in an anonymized database.

### 2.3 Sample collection

Serum and cerebrospinal fluid (CSF) samples were obtained as part of the diagnostic workup by trained neurologists who conducted lumbar punctures before the administration of corticosteroids and DMT. Laboratory staff aliquoted and stored them at –80 °C until further processing.

### 2.4 OCB analysis, kappa index, IgG index quantification, and antibody determination

Oligoclonal bands (OCB) in serum and cerebrospinal fluid (CSF) were assessed using isoelectric focusing (IEF) followed by immunoblotting, in accordance with previously established protocols (26). A positive OCB result was defined by the presence of two or more IgG bands in the CSF that were absent in the corresponding serum sample.

Kappa free light chains (KFLCs) were quantified by turbidimetry in an Optilite turbidimeter using Optilite Freelite Mx Kappa FreeKits (The Binding Site, Birmingham, UK). The kappa index was determined using the formula: [CSF KFLC (mg/dL)/serum KFLC (mg/dL)]/[CSF albumin (mg/dL)/serum albumin (mg/dL)]. There are different strategies for dealing with patients with undetectable levels of CSF KFLC (27, 28). In our study, we decided to exclude them from the statistical analysis (n = 33).

Some publications state that the use of a Reiber diagram for kappa free light chain analysis could improve the performance of the kappa index (29). We also determined ITGS using the Reiber diagram and Reiber’s formulae, where Q_lim_ represents the upper physiological limit of the CSF/serum quotient for KFLC, Q_low_ is the lower physiological limit, and Q_mean_ is the theoretical mean curve between both limits according to the Reiber model. Formulae were calculated as previously described (13).

IgG was quantified by nephelometry in an Atellica Neph 630 nephelometer using N Antiserum to Human IgG kits. The IgG index was determined using the formula: [CSF IgG (mg/dL)/serum IgG (mg/dL)]/[CSF albumin (mg/dL)/serum albumin (mg/dL)]. We considered IgG index values above 0.7 to be positive.

For antibodies IgG anti–aquaporin-4 (AQP4) and anti–myelin oligodendrocyte glycoprotein (MOG), serum samples were analyzed using the IIFT: NMOSD Screen 1 kit (EUROIMMUN, Lübeck, Germany), which detects these antibodies by indirect immunofluorescence in fixed transfected cells. Antibody testing was performed in all patients presenting with optic neuritis, myelitis, or brainstem syndromes whenever clinical or MRI features were atypical for MS or raised suspicion of neuromyelitis optica spectrum disorder (NMOSD)/myelin oligodendrocyte glycoprotein antibody-associated disease (MOGAD).

### 2.5 MRI study

MRI studies were performed at baseline using a 1.5T or 3T magnet, capturing a range of sequences, including transverse spin-echo proton-density weighted and/or T2-weighted spin-echo sequences, in addition to transverse and sagittal T2-fluid-attenuated inversion recovery sequences. Brain scans also encompassed transverse T1-weighted spin-echo before and after contrast injection. The obtained spinal cord sequences included T2-weighted fast spin-echo and sagittal short-tau inversion recovery. Gadolinium-enhanced sagittal T1-weighted and axial T2-weighted fast spin-echo were performed, covering segments with confirmed or suspected lesions identified in the sagittal sequences.

All brain MRIs were performed using the MAGNIMS MRI protocol, which involves acquiring a 1-mm 3D FLAIR scan, allowing for reconstructions in all three planes (axial, sagittal, and coronal).

### 2.6 Statistical analysis

Categorical variables were described using absolute and relative frequencies, while continuous variables were presented as medians with interquartile ranges (IQRs).

The kappa index medians among the three categories (MS, CIS, and NMOSD/MOGAD) were compared using the Kruskal-Wallis one-way ANOVA test with a Dunn’s post-hoc test.

To calculate sensitivity, specificity, positive predictive value (PPV), and negative predictive value (NPV) for all tests, we defined true positives (TPs) as patients with positive results for any of the three variables who were subsequently diagnosed with MS. We considered true negatives (TNs) those with a negative result for any of the three tests who did not receive an MS diagnosis during follow-up. False positives (FPs) were defined as patients who did not receive an MS diagnosis during follow-up but had positive results for any variable. False negatives (FNs) were defined as patients with MS who had negative results for the kappa index, OCB, or IgG index.

Sensitivity, specificity, positive predictive value (PPV), and negative predictive value (NPV) were calculated using the following formulas:

- Sensitivity: (TP/[TP + FN]) x 100
- Specificity: (TN/[TN + FP]) x 100
- PPV: (TP/[TP + FP]) x 100
- NPV: (TN/[TN + FN]) x 100

We employed the McNemar test to compare sensitivity and specificity between the methods.

We performed a Cox regression to assess whether the kappa index value could affect the time to MS diagnosis. For comparing the survival curves, we used a Mantel-Cox test.

All analyses were performed using Stata 17 (StataCorp, College Station, TX, USA) or GraphPad Prism 9.5.1 statistical packages. All tests were two-tailed, and p-values below 0.05 were considered significant.

### 2.7 Data availability

Original data will be available to any researcher in the field for three years upon request to the corresponding author.

## 3 Results

### 3.1 Clinical, radiological, and epidemiological variables

A total of 391 patients with CIS and available CSF/serum samples were selected. Baseline clinical and demographic data for the cohort are shown in **Table 1**. CSF data are shown in **Table 2**. The diagnosis of MS was established using the 2017 McDonald criteria.

**Table 1:** Clinical and demographic variables.

Variable	Patients (n = 391)
Patient age, years [Median (IQR)]	35.0 (26.8–44.4)
Sex [number of women/men]	250/141
Patients with confirmed MS during follow-up. [Number (%)]	328 (83.9%)
Time to lumbar puncture, months [median (IQR)]	4.3 (1.5–8.3)
Time of follow-up of non-MS patients (years) [median (IQR)]	7.0 (5.4–8.3)
Time to conversion to MS (months) [median (IQR)]	27.4 (8.4–56.3)
Regions with demyelinating lesions [Number (%)]	
0	24 (6.1%)
1	28 (7.2%)
≥2	339 (86.7%)
CIS topography [Number (%)]	
Optic nerve	84 (21.5%)
Brainstem	78 (20.0%)
Spinal cord	165 (42.2%)
Cerebral hemisphere	54 (13.8%)
Multifocal	10 (2.6%)
T2 lesions at baseline [Number (%)]	
No lesions observed	24 (6.1%)
1–3	63 (16.1%)
4–9	98 (25.1%)
≥10	206 (52.7%)

IQR, interquartile range; MS, multiple sclerosis; CIS, clinically isolated syndrome.

**Table 2:** Cerebrospinal fluid (CSF) data.

CSF data	Patients (n = 391)
Albumin index [median (IQR)]	4.44 (3.32–5.88)
Number of leukocytes (cells/µL) [median (IQR)]	4.0 (2.0–8.0)
Glycorrhachia (mg/dL) [median (IQR)]	56.00 (52.0–59.25)
Proteinorrhachia (mg/dL) [median (IQR)]	25.00 (18.80–32.57)

IQR, interquartile range; CSF, cerebrospinal fluid.

Based on the topography of the first relapse, our cohort included 84 patients with optic nerve CIS (21.5%), 78 with brainstem CIS (20.0%), 165 with spinal cord CIS (42.0%), 54 with cerebral hemisphere involvement (13.8%), and 10 with multifocal manifestations (2.6%). We did not observe differences between the optic nerve group and the other CIS topographies in terms of age, sex, time of follow-up, the number of T2 lesions, or the presence of OCB.

**Figure 1:**
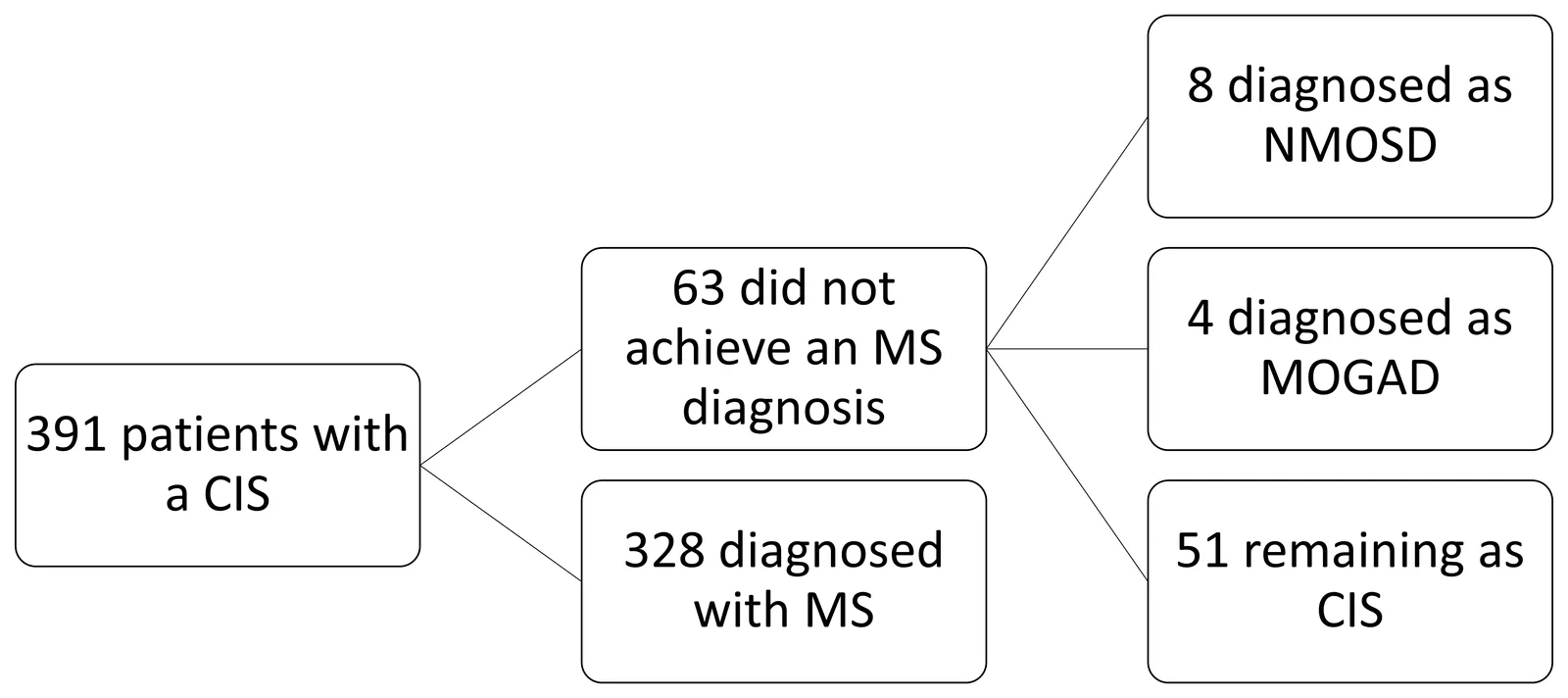
Flowchart. From our initial cohort of 391 patients with clinically isolated syndrome (CIS), 328 were later diagnosed with multiple sclerosis (MS) and 63 were not. Among the latter, 51 remained with CIS, eight were diagnosed with neuromyelitis optica spectrum disorder (NMOSD), and four with MOG-antibody–associated disease (MOGAD).

A total of 328 of the 391 patients had a confirmed MS diagnosis (based on the 2017 McDonald criteria) during follow-up. Of the remaining 63 patients, 8 received a diagnosis of neuromyelitis optica spectrum disorder (NMOSD) (4 seropositive and 4 seronegative for anti-aquaporin-4 antibodies), and 4 received a diagnosis of myelin oligodendrocyte glycoprotein antibody-associated disease (MOGAD). The remaining 51 remained with CIS during follow-up (**Figure 1**). All patients with a final diagnosis of MOGAD or NMOSD were negative for OCB, but two of the eight patients with NMOSD showed kappa index values of 8.24 and 8.85 (both patients had autoantibodies against aquaporin-4). In all, 33 patients showed CSF kappa values below the detection limit of the technique. Of these, 32 were patients remaining as CIS during the study, and one was a patient with an MS diagnosis during follow-up. They were excluded from subsequent statistical analyses.

Kappa index values were higher in patients who received an MS diagnosis (43.5 [16.9–112.3], median [25%–75% IQR]) compared with those remaining with CIS (2.8 [0.9–3.4, p < 0.0001), or those with NMOSD/MOGAD (5.7 [4.1–5.9], p < 0.0001). We did not find significant differences between the patients remaining with CIS and the NMOSD/MOGAD groups.

**Figure 2:**
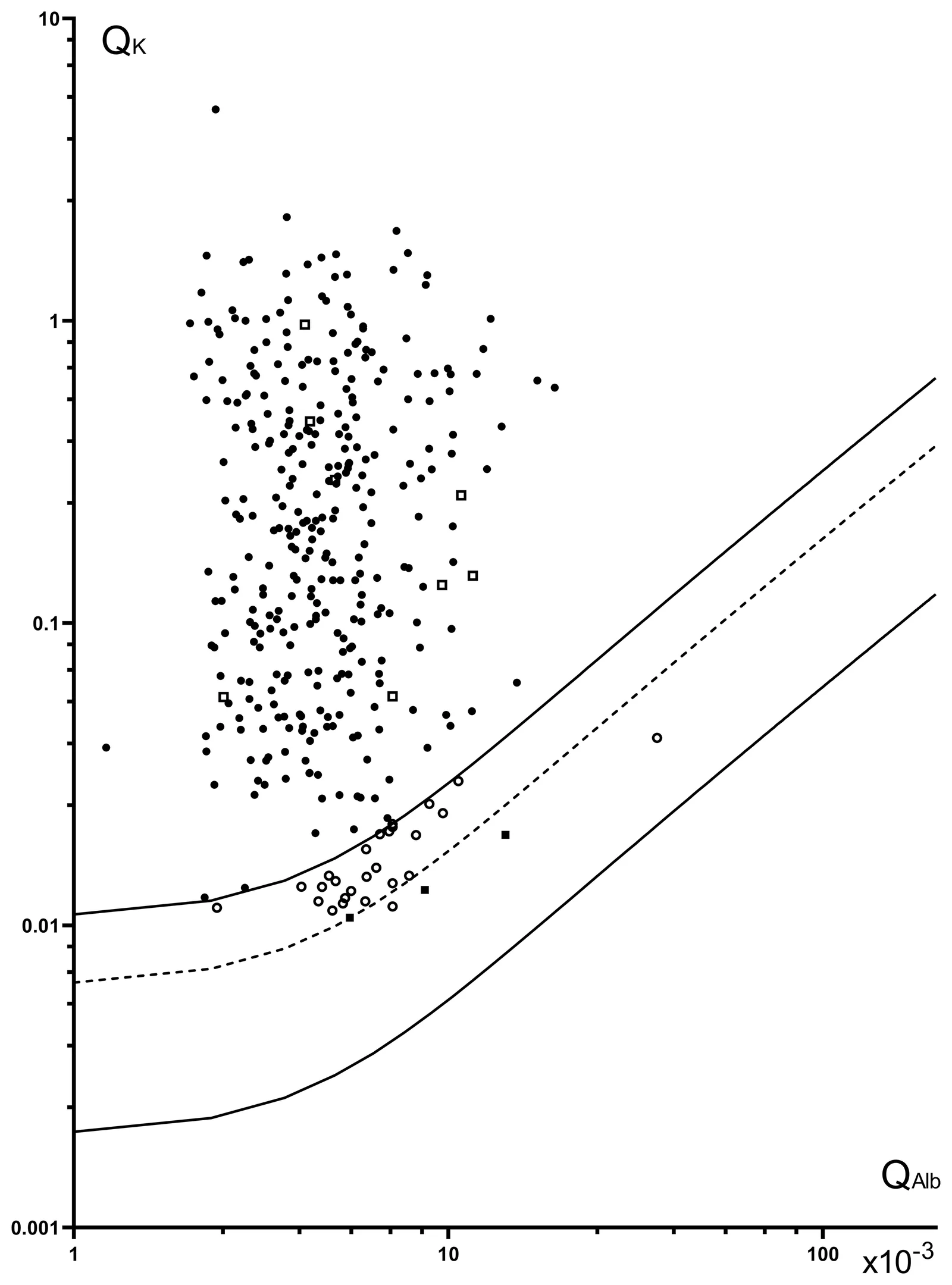
The plot illustrates the relationship between the cerebrospinal fluid/serum albumin quotient (Q_Alb_) and the cerebrospinal fluid/serum κ free light chain quotient (Q_kappa_) on a double logarithmic scale. Each dot represents an individual patient value. The solid lines delineate the upper and lower boundaries of the physiological diffusion range according to the Reiber hyperbolic model, while the dashed line indicates the theoretical mean curve. Increasing Q_kappa_ values with higher Q_Alb_ reflect progressive impairment of the blood-brain barrier. Data points above the upper limit line correspond to intrathecal synthesis of kappa free light chains. Among patients with positive Reiber kappa values (RKV), 319 of 322 patients had an MS diagnosis, and 8 of 36 patients who remained with CIS or received a diagnosis of NMOSD or MOGAD were positive (7 had CIS, and 1 received a diagnosis of NMOSD).

Results of the Reiber diagram are shown in **Figure 2**. Every Q_kappa_ value above Q_lim_ was considered positive. Of the patients with positive Reiber kappa values (RKV), 319 of 322 patients with an MS diagnosis and 8 of 36 patients remaining with CIS or diagnosed with NMOSD or MOGAD were positive (7 were CIS patients, and the other received a diagnosis of NMOSD).

The median follow-up time for the 51 patients who remained with CIS was 7.0 [5.4–8.3] years, with only six patients followed for less than five years. These six patients were all negative for OCB, showed kappa index values below 6.1, had IgG index values below 0.7, and had negative Reiber kappa values (RKV). Follow-up time is included in **Table 1**.

### 3.2 Sensitivity, specificity, positive predictive value, and negative predictive value of different tests

**Figure 3:**
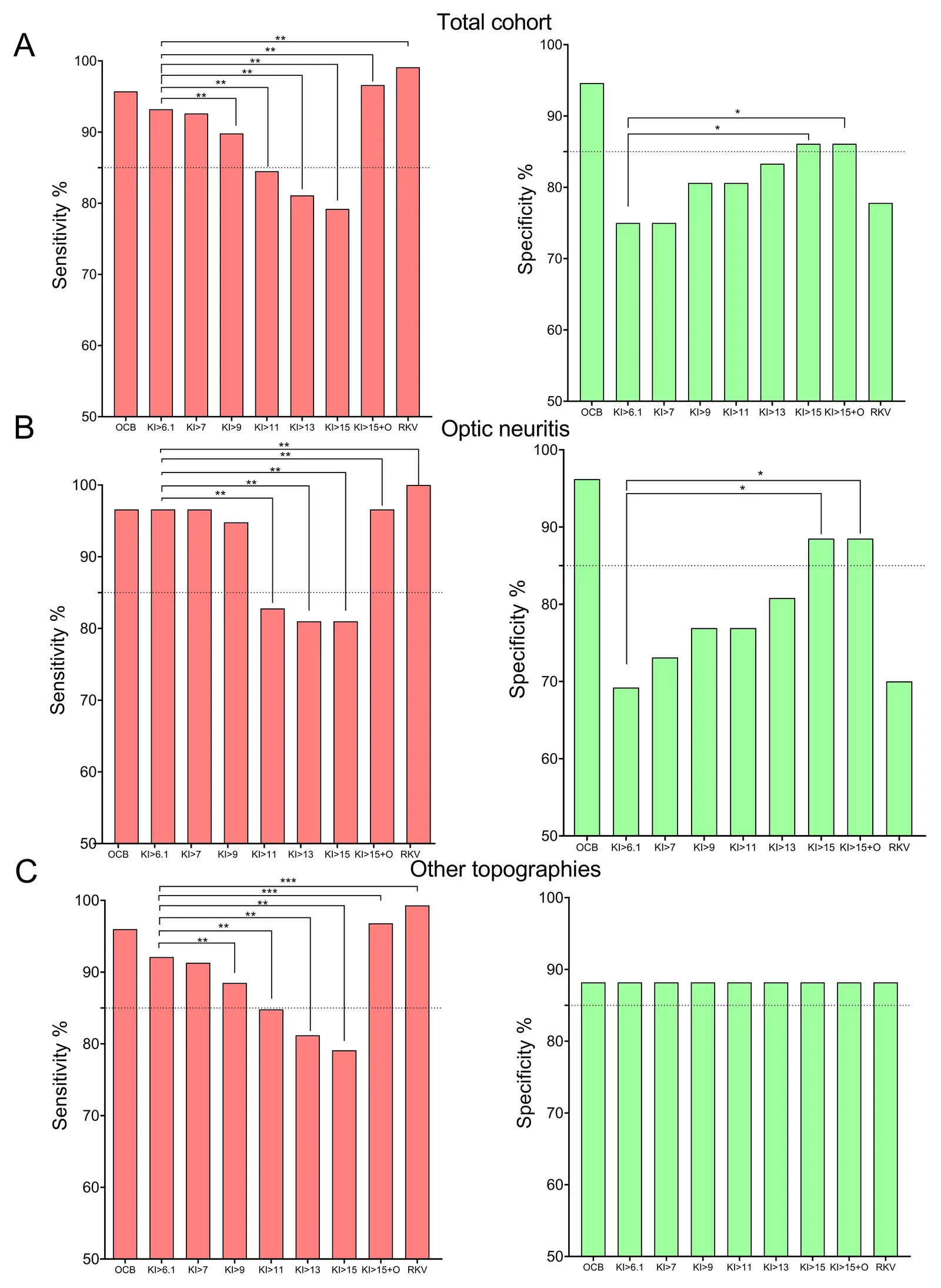
In this figure, we illustrate the comparison in sensitivity and specificity among three cohorts: the overall cohort **(A)**, patients with optic neuritis **(B)**, and patients with other clinical topographies **(C)**. In every cohort, Reiber Kappa Values (RKV) yielded the highest sensitivity, while Oligoclonal IgG Bands (OCB) showed the highest specificity. Regarding to the kappa index (KI), the figure shows how in the CIS cohort that does not include optic neuritis, the specificity of the KI remains the same in every tested cut-off value. However, in the case of optic nerve CIS patients, increasing the cut-off values and combining the kappa index with OCB (KI>15+O) improves specificity and sensitivity.

We evaluated the sensitivity, specificity, positive predictive value (PPV), and negative predictive value (NPV) of different kappa index cut-off values, oligoclonal bands (OCB), the IgG index, and RKV. Regarding the kappa index, we assessed cut-off values ranging from 6.1 to 20, increasing in one-unit increments. Because sensitivity and specificity remained relatively stable across this interval, we report results for every second value up to a cut-off of 15. Cut-off values above 15 did not improve specificity and were associated with a marked reduction in sensitivity. Results are shown in **Figure 3** and in **Supplementary Table 2**.

The IgG index showed low sensitivity (66.8%) and specificity (77.8%).

The Reiber diagram yielded the highest sensitivity for detecting ITGS, at 99.1%, with a specificity of 77.8%. OCB showed a sensitivity of 95.7% and a specificity of 91.7%.

When exploring the kappa index, the highest sensitivity was observed for a cut-off value of 6.1 (93.2%), although specificity at this threshold was 75.0% (similar to the 77.8% shown by RKV). Specificity increased progressively with higher cut-offs, reaching significant differences at a cut-off of 15, which yielded a specificity of 86.1%. Conversely, sensitivity decreased significantly at cut-off values of 9 or higher (**Figure 3A**, **Supplementary Tables 2**, **3**).

We next examined whether combining the kappa index with OCB could improve sensitivity. We selected a cut-off of 15 for this analysis, as it provided the best specificity. Samples were classified as positive if either OCB or a kappa index ≥ 15 was present, and as negative if the kappa index was < 15 and OCB was absent. Using this algorithm, sensitivity increased to 96.6%, a value higher than that obtained with any individual kappa index cut-off (**Figure 3A**; **Supplementary Tables 2**, **3**). This improvement reflects the lower concordance between the kappa index and OCB in patients with kappa index values < 15. Indeed, among 260 patients with a kappa index ≥ 15, 256 (98.5%) were OCB-positive, whereas only 53 of the 98 patients with a kappa index < 15 were OCB-negative (54.1%). Specificity remained at 86.1% with the combined approach. These findings indicate that a cut-off of 15, when supplemented by OCB testing, optimizes both sensitivity and specificity.

**Figure 4:**
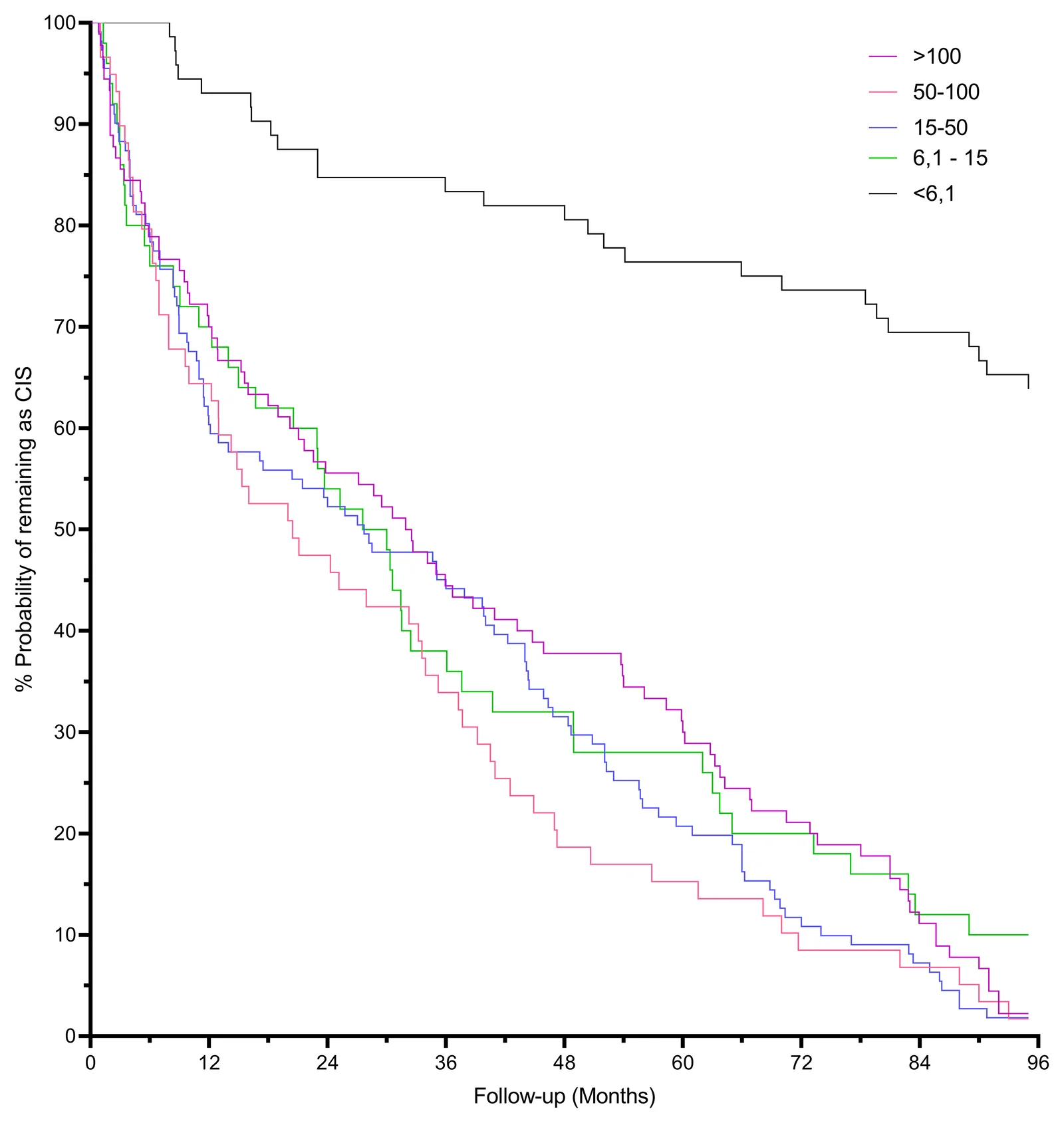
Cox regression. In this graph, we compare the MS conversion curves of patients classified based on their kappa index values. This graph shows that only those patients with a kappa index below 6.1 showed significant differences in time to MS conversion.

We also investigated, since the kappa index is a quantitative biomarker, whether its magnitude could influence the time to conversion to MS. Patients were stratified into five groups based on kappa index values (<6.1, 6.1–15, 15–50, 50–100, and >100). Significant differences were observed only between each group and the group with a kappa index < 6.1. These results are shown in **Figure 4**; **Table 3**.

**Table 3:** Hazard ratios, confidence intervals, and p-values from the Cox regression analysis for groups of patients classified by their kappa index values.

Curves tested	Hazard ratio (95% CI)	P-values
<6.1 vs. 6.1–15	4.73 (2.80–8.00)	<0,0001
<6.1 vs. 15–50	1.21 (0.86–1.69)	<0,0001
<6.1 vs. 50–100	6.06 (3.74–9.82)	<0,0001
<6.1 vs. >100	5.43 (3.72–7.92)	<0,0001
6.1–15 vs. 15–50	1.20 (0.86–1.69)	0,2836
6.1–15 vs. 50–100	1.32 (0.90–1.96)	0,1572
6.1–15 vs. >100	1.05 (0.74–1.51)	0,7670
15–50 vs. 50–100	1.13 (0.82–1.56)	0,4516
15–50 vs. >100	0.82 (0.62–1.01)	0,1684
50–100 vs. 100	0.77 (0.54–1.01)	0,1094

CI, confidence interval.

For a deeper analysis of the data, we assessed whether the CIS topography affected the performance of different kappa index cut-offs. We observed distinct patterns in optic neuritis compared with other CIS locations (brainstem, spinal cord, cerebral hemisphere, and multifocal presentations). Results are shown in **Figures 3B, C**; **Supplementary Tables 4**-**7**.

In optic neuritis, findings mirrored those of the overall cohort. Again, the optimal balance of sensitivity and specificity was achieved using OCB, although a cut-off of 15 combined with OCB testing yielded good sensitivity and specificity values (**Figure 3B**; **Supplementary Tables 4**, **5**).

The remaining CIS locations differed from optic neuritis but showed similar results among themselves (**Supplementary Tables 8**-**11**) and were therefore analyzed together (**Figure 3C**; **Supplementary Tables 6**, **7**). In these topographies, a kappa index cut-off of 6.1 produced sensitivity (92.1%) and specificity (88.2%) comparable to those of OCB detection (96.0% and 88.2%). Higher cut-offs did not significantly increase specificity but did significantly reduce sensitivity. Combining OCB with the kappa index did not yield significant improvements over the 6.1 cut-off (**Figure 3C**). Sensitivity was even higher when applying the Reiber formulae, reaching 99.3%, while specificity remained at 88.2%.

### 3.3 Algorithm for the use of the kappa index and OCB based on CIS topography

**Figure 5:**
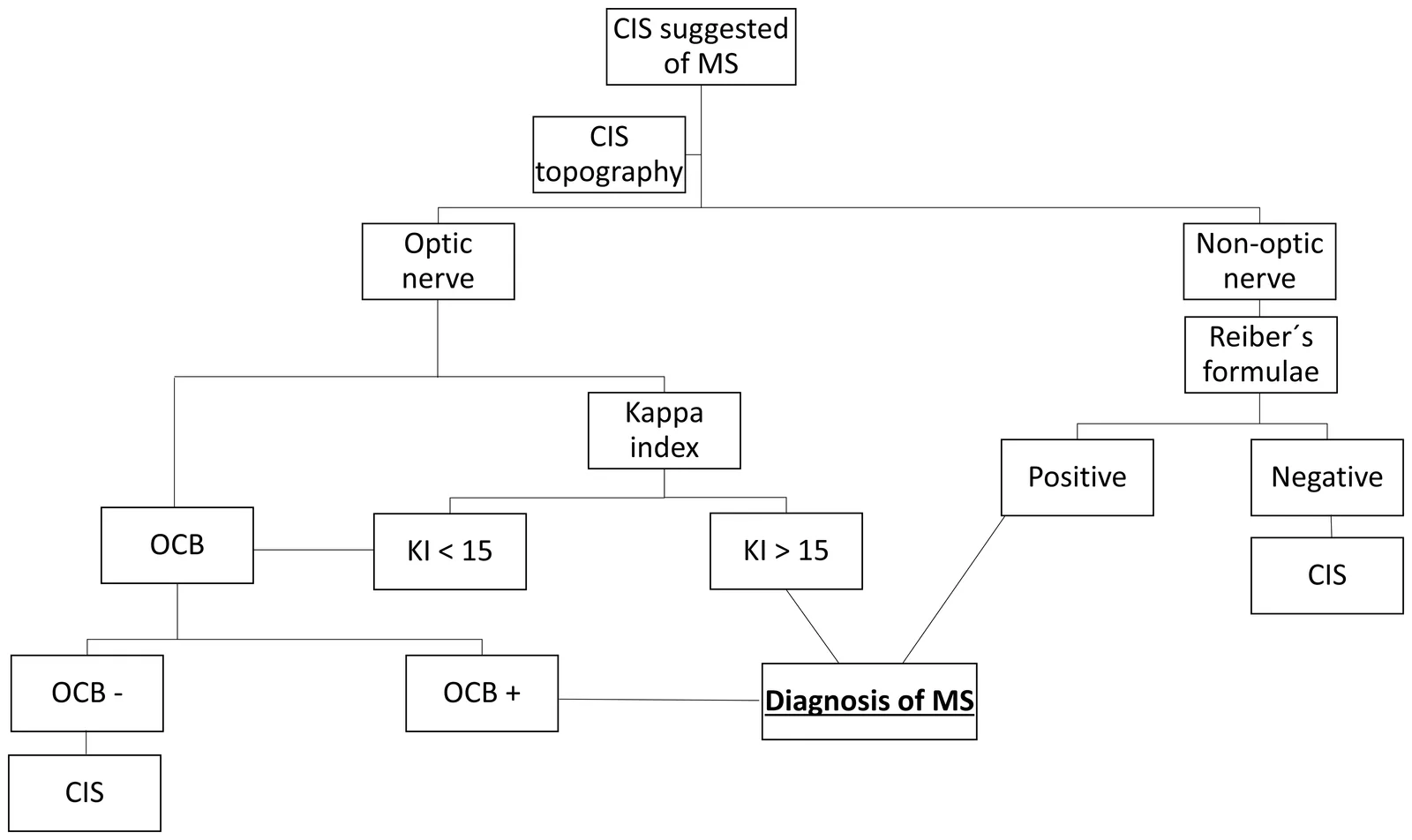
Diagnostic algorithm for patients with CIS suggestive of MS. The diagnostic algorithm for patients with CIS suggestive of MS involves first classifying the CIS type based on clinical and radiological data. If the CIS type is optic nerve, either OCB or the kappa index with a cut-off value of 15 can be used to determine CSF positivity. If the kappa index is below 15, OCB testing should be performed to rule out MS. On the other hand, regarding non-optic nerve CIS, the Reiber diagram shows the highest sensitivity and a specificity comparable to that of OCB and a kappa index cut-off value of 6.1. CIS, clinically isolated syndrome; KI, kappa index; MS, multiple sclerosis; OCB, oligoclonal bands.

Based on the diagnostic performance of both techniques for discriminating MS, we propose an algorithm that combines the RKV, the kappa index, and OCB in patients presenting with a typical CIS, adapted to the CIS topography (**Figure 5**).

For patients with optic neuritis CIS, two approaches can be used to rule out MS: either performing the OCB test or applying a kappa index cut-off of 15. When using the kappa index, values above 15 indicate CSF positivity. If the kappa index is below 15, OCB testing should be performed to complete the evaluation.

For patients with any other CIS phenotype, a positive RKV is sufficient to determine CSF positivity without the need for additional OCB testing. RKV could be substituted with a kappa index cut-off of 6.1, although it gives lower sensitivity.

It is essential to emphasize that both the kappa index and OCB are components of a broader diagnostic process. Clinical and MRI follow-up remain necessary to establish a definitive diagnosis of MS.

## 4 Discussion

The new 2024 McDonald criteria (30) included the kappa index as an additional diagnostic tool to demonstrate ITGS. Due to the technical complexity of the OCB technique, different methods that are easy, reproducible, and accessible to non-specialized laboratories have been proposed (7). These methods include the Reiber formula for KFLC, the IgG and the kappa index. Previous studies showed that the IgG index could not constitute a substitute for OCB, due to its lack of sensitivity (8). However, the kappa index showed better results in several studies (9, 16, 17), although it showed less specificity than OCB (18), probably because KFLC levels could be elevated in other inflammatory diseases (19).

KFLC assessment and OCB should not be seen as competing biomarkers but rather as complementary indicators of intrathecal immunoglobulin synthesis. Taken together, the integration of both biomarkers may enhance diagnostic accuracy in early multiple sclerosis and related inflammatory disorders, supporting their complementary use in contemporary diagnostic frameworks (31).

With all these considerations, the kappa index was included in the criteria. Although the criteria suggested the use of the 6.1 cut-off, others were proposed, usually ranging between 6 and 12, aiming to increase the specificity of this technique (16, 17, 26, 32). The value of 6.1 was established as useful in different research studies, but its usefulness remains to be demonstrated in real clinical practice (17). Other formulae to explore KFLC synthesis have also been explored. In this regard, it has been reported that the Reiber diagram for KFLC shows higher sensitivity than the kappa index for detecting intrathecal synthesis of KFLC (29).

When exploring the sensitivity and specificity of the different algorithms for detecting intrathecal KFLC, the literature shows different strategies for dealing with patients with undetectable CSF KFLC levels (27, 28). First, it is important to consider the technique used for KFLC detection. There are two methods based on nephelometry or turbidimetry. Both rely on distinct optical detection principles. Nephelometry measures light scattered at an angle by immune complexes, whereas turbidimetry quantifies the reduction in transmitted light caused by particle formation. As a consequence of these methodological differences, turbidimetric assays generally achieve substantially lower limits of detection (26). We used a turbidimetric method in our study to reduce the number of patients with undetectable values. Despite this, it is necessary to consider how to manage patients who still have undetectable CSF KFLC levels. Different approaches have been studied, including assigning the lower detection limit of the technique, half of this value, or zero to these patients, or, to avoid false assumptions, excluding them from sensitivity and specificity calculations (27, 28). This was the option adopted in this work.

The diagnostic performance of a test for MS can be assessed in two ways: in the general population or in a group of patients at risk (CIS patients). Both approaches have been used in the literature, and both have different advantages and disadvantages (33, 34). Including only patients at risk could exclude patients with other inflammatory diseases different from MS that can give false-positive results. However, it allows us to assess the effect of other variables, such as CIS location, which can be relevant for the analysis. The first approach has already been addressed in previous studies that have provided data on the best cut-off to perform a differential diagnosis between MS and other inflammatory neurological diseases (27, 35). We chose the second approach. This is also a real-life approach since we included CIS patients defined clinically, with no support from any other paraclinical test. We think this approach is relevant since it contributes to identifying the best combination of biomarkers for the early identification of CIS patients who will receive an MS diagnosis, including clinical data such as CIS location. This approach can also contribute to exploring the value of the kappa index in the differential diagnosis of other demyelinating diseases such as MOGAD and NMOSD (36). This is important because although both have their own diagnostic biomarkers, some patients with NMOSD can be negative for anti-aquaporin antibodies, and some MS patients can have anti-MOG antibodies (37).

Moreover, recent evidence from neuropsychiatric research shows that CSF inflammatory markers and kappa free light chains may also be relevant beyond classical demyelinating disorders, as inflammatory changes have been detected in conditions such as paranoid schizophrenia (38) and in psychiatric syndromes associated with neuronal or paraneoplastic autoantibodies (39). These findings reinforce the idea that KFLC and related CSF biomarkers could have broader diagnostic potential across a wider spectrum of neuroinflammatory and neuropsychiatric diseases, further supporting the need to refine their use in early and differential diagnosis of these conditions.

In this study, 391 CIS patients were evaluated to assess the diagnostic performance of different kappa index cut-off values. Raising the cut-off value to 15 resulted in an improved specificity (86.1%) compared with the recommended cut-off and the Reiber diagram. Furthermore, combining a kappa index cut-off of 15 with oligoclonal bands (OCB) increased sensitivity to 96.6%, without compromising specificity. These findings indicate *a priori* that raising the kappa index cut-off enhances the diagnostic accuracy for MS and supports the use of combined criteria for greater clinical reliability.

However, differences emerged when we classified CIS patients according to their topography. The OCB test alone or in combination with a kappa index of 15 was the best option for patients with optic neuritis. They improved the specificity, sensitivity, NPV, and PPV compared with other strategies. A previous study assessing the value of the kappa index in optic neuritis (40) also demonstrated the utility of higher cut-off values for the differential diagnosis of MS, especially to differentiate MS from NMOSD and from optic neuritis patients not receiving an MS diagnosis. Oligoclonal IgG bands also showed a high sensitivity and specificity for predicting MS diagnosis in patients with optic neuritis (41).

On the other hand, when considering other CIS topographies, Reiber’s formulae achieved the best results in sensitivity, with specificity comparable to that of any kappa index cut-off and to OCB. This is consistent with the physiological foundation of the Reiber model, which adjusts KFLC concentrations to the individual albumin quotient and therefore better accounts for patient-specific CSF/serum dynamics (42, 43).

These differences between optic nerve CIS patients and the other topographies could be due to the lack of dedicated sequences for detecting optic nerve lesions in MS compared with the other territories (44). The consideration of the optic nerve as the fifth territory in the new MS diagnostic criteria will probably improve this (45). The value of CIS location in establishing the best cut-off values for the kappa index in MS diagnosis will be confirmed in future studies.

A global, multicenter initiative is currently underway to harmonize methodological and clinical aspects of KFLC and OCB assessment (46), which is expected to address unresolved questions regarding assay variability, cut-off standardization, and diagnostic implementation.

This study has some limitations. It is a single-center study that includes some retrospectively collected data. The subgroup of CIS patients diagnosed with conditions other than multiple sclerosis was relatively small. Two different MRI field strengths (1.5T and 3T) were used. The kit used for anti-AQP4 and anti-MOG detection, based on fixed transfected cells, shows lower sensitivity than live-cell-based assays. In addition, the lack of systematic testing in all patients with myelitis or brainstem presentations could theoretically allow misclassification of a small number of NMOSD/MOGAD cases.

In conclusion, this study shows that KFLC assessment and OCB can complement each other to improve MS diagnosis.

## Data Availability

The raw data supporting the conclusions of this article will be made available by the authors, without undue reservation.
