# Supplementary material for: Kappa index: real-world study for assessing cut-off values that provide the best accuracy for multiple sclerosis diagnosis in patients with CIS
Source: Front Immunol. 2026 Aug 25;17:1771566. doi: 10.3389/fimmu.2026.1771566 (PMC13549829; doi:10.3389/fimmu.2026.1771566)
Supplement: Supplementary file 1 [file Table1.docx]

**Supplementary table 1.** In this supplementary table we show all the cut-offs that we tried, with their sensitivities, specificities positive predictive values and negative predictive values, alongside with the confidence intervals.

| TEST | Sensitivity (95% CI) | Specificity (95% CI) | PPV (95% CI) | NPV (95% CI) |
| --- | --- | --- | --- | --- |
| OCB | 95.7% (93.2% - 97.6%) | 91,7% (93,2% - 97,6%) | 99.0% (97.2% - 99.7%) | 71,7% (57,4% - 82,7%) |
| IgG index | 66,8% (61,4% - 71,7%) | 77,8% (61,9% - 88,3%) | 97,6% (95,2% - 98,8%) | 22,5% (16,3% - 30.1%) |
| Reiber´s KFLC | 99,1% (97,3% - 99,8%) | 77,8% (61,9% - 88,3%) | 97,6% (95,2% - 98,8%) | 90,3% (75,1% - 96,7%) |
| Kappa index ≥ 6.1 | 93,2% (89,9% - 95,5%) | 75,0% (58,9% - 86,3%) | 97,1% (94,6% - 98,5%) | 55,1% (41,3% - 68,2%) |
| Kappa index ≥ 7 | 92,6% (89,2% - 94,9%) | 75,0% (58,9% - 86,3%) | 97,1% (94,6% - 98,5%) | 52,9% (39,5% - 66,0%) |
| Kappa index ≥ 8 | 91.0% (87,4% - 93,7%) | 75,0% (58,9% - 86,3%) | 97,0% (94,4% - 98,4%) | 48,2% (35,7% - 61,0%) |
| Kappa index ≥ 9 | 89,8% (86,0% - 92,6%) | 80,6% (65,0% - 90,3%) | 97,6% (95,2% - 98,9%) | 46,8% (34,9% - 59,0%) |
| Kappa index ≥ 10 | 86,7% (82,5% - 89.9%) | 80,6% (65,0% - 90,3%) | 97,6% (95,0% - 98,8%) | 40,3% (29,7% - 51,8%) |
| Kappa index ≥ 11 | 84,5% (80,1% - 88,0%) | 80,6% (65,0% - 90,3%) | 97,5% (94,9% - 98,8%) | 36,7% (26,9% - 47.7%) |
| Kappa index ≥ 12 | 82,9% (78,4% - 86,6%) | 77,8% (61,9% - 88,3%) | 97,1% (94,4% - 98,5%) | 33,7% (24,5% - 44,4%) |
| Kappa index ≥ 13 | 81,1% (76,4% - 85,0%) | 83,3% (68,1% - 92,1%) | 97,8% (95,2% - 99.0%) | 33.0% (24,2% - 43,1%) |
| Kappa index ≥ 14 | 79,2% (74,4% - 83,3%) | 83,3% (68,1% - 92,1%) | 97,7% (95,1% - 98,9%) | 30,9% (22,6% - 40,7%) |
| Kappa index ≥ 15 | 79,2% (74,4% - 83,3%) | 86,1% (71,3% - 93,9%) | 98,1% (95,6% - 99,2%) | 31,6% (23,3% - 41,4%) |
| Kappa index ≥ 16 | 76,4% (71,5% - 80,7%) | 86,1% (71,3% - 93,9%) | 98,0% (95,4% - 99,2%) | 29,0% (21,2% - 38,2%) |
| Kappa index ≥ 17 | 74,5% (69,5% - 79,0%) | 86,1% (71,3% - 93,9%) | 98,0% (95,3% - 99,1%) | 27,4% (20,1% - 36,3%) |
| Kappa index ≥ 18 | 73,3% (68,2% - 77,8%) | 86,1% (71,3% - 93,9%) | 97,9% (95,2% - 99,1%) | 26,5% (19,3% - 35,2%) |
| Kappa index ≥ 19 | 71,7% (66,6% - 76,4%) | 86,1% (71,3% - 93,9%) | 97,8% (95,14% - 99,1%) | 25,4% (18,5% - 33,8%) |
| Kappa index ≥ 20* | 70,5% (65,3% - 75,2%) | 86,1% (71,3% - 93,9%) | 97,8% (95,06% - 99,1%) | 24,6% (17,9% - 32,8%) |
| Kappa index ≥ 15 or OCB+ | 96,6% (94,0% - 98,1%) | 86,1% (71,3% - 93,9%) | 98,4% (96,4% - 99,3%) | 73,8% (58,9% - 84,7%) |

Note: *increasing cut-off values above 15 did not improve specificity but decreased the sensitivity remarkably.

Abbreviations: CI, confidence interval; PPV, positive predictive value; NPV, negative predictive value; OCB, oligo-clonal bands; KFLC, kappa free light chains.

**Supplementary table 2.** Sensitivity, specificity, positive predictive value and negative predictive value of kappa index, IgG index, Reiber for kappa free light chains and oligoclonal IgG bands and their combinations

| Test | Sensitivity (95% CI) | Specificity (95% CI) | PPV (95% CI) | NPV (95% CI) |
| --- | --- | --- | --- | --- |
| OCB | 95.7% (93.2% - 97.6%) | 91,7% (93,2% - 97,6%) | 99.0% (97.2% - 99.7%) | 71,7% (57,4% - 82,7%) |
| IgG index | 66,8% (61,4% - 71,7%) | 77,8% (61,9% - 88,3%) | 97,6% (95,2% - 98,8%) | 22,5% (16,3% - 30.1%) |
| Reiber´s KFLC | 99,1% (97,3% - 99,8%) | 77,8% (61,9% - 88,3%) | 97,6% (95,2% - 98,8%) | 90,3% (75,1% - 96,7%) |
| Kappa index ≥ 6.1 | 93,2% (89,9% - 95,5%) | 75,0% (58,9% - 86,3%) | 97,1% (94,6% - 98,5%) | 55,1% (41,3% - 68,2%) |
| Kappa index ≥ 7 | 92,6% (89,2% - 94,9%) | 75,0% (58,9% - 86,3%) | 97,1% (94,6% - 98,5%) | 52,9% (39,5% - 66,0%) |
| Kappa index ≥ 9 | 89,8% (86,0% - 92,6%) | 80,6% (65,0% - 90,3%) | 97,6% (95,2% - 98,9%) | 46,8% (34,9% - 59,0%) |
| Kappa index ≥ 11 | 84,5% (80,1% - 88,0%) | 80,6% (65,0% - 90,3%) | 97,5% (94,9% - 98,8%) | 36,7% (26,9% - 47.7%) |
| Kappa index ≥ 13 | 81,1% (76,4% - 85,0%) | 83,3% (68,1% - 92,1%) | 97,8% (95,2% - 99.0%) | 33.0% (24,2% - 43,1%) |
| Kappa index ≥ 15 | 79,2% (74,4% - 83,3%) | 86,1% (71,3% - 93,9%) | 98,1% (95,6% - 99,2%) | 31,6% (23,3% - 41,4%) |
| Kappa index ≥ 15 or OCB+ | 96,6% (94,0% - 98,1%) | 86,1% (71,3% - 93,9%) | 98,4% (96,4% - 99,3%) | 73,8% (58,9% - 84,7%) |

Abbreviations: CI; confidence interval; PPV, positive predictive value; NPV, negative predictive value; OCB, oligo-clonal bands; KFLC, kappa free light chains.

**Supplementary table 3**. Statistical differences in sensitivity and specificity in the cohort. In terms of specificity, only a kappa index cut-off of 15—and its combination with OCB—significantly improved specificity compared to the conventional cut-off value of 6.1.

| **McNemar** | Kappa index ≥ 6.1 | Kappa index ≥ 7 | Kappa index ≥ 9 | Kappa index ≥ 11 | Kappa index ≥ 13 | Kappa index ≥ 15 | Kappa index ≥ 15 or OCB+ |
| --- | --- | --- | --- | --- | --- | --- | --- |
| **SENSITIVITY** | | | | | | | |
| Kappa index ≥ 6.1 |  | 0.5 | **0.001↓** | **0.001↓** | **<0.001↓** | **<0.001↓** | **0.008↑** |
| Kappa index ≥ 7 |  |  | **0.004↓** | **0.004↓** | **<0.001↓** | **<0.001↓** | **0.003↑** |
| Kappa index ≥ 9 |  |  |  | >0.99 | **0.002↓** | **<0.001↓** | **<0.001↑** |
| Kappa index ≥ 11 |  |  |  |  | **0.002↓** | **<0.001↓** | **<0.001↑** |
| Kappa index ≥ 13 |  |  |  |  |  | **<0.001↓** | **<0.001↑** |
| Kappa index ≥ 15 |  |  |  |  |  |  | **<0.001↑** |
| **SPECIFICITY** | | | | | | | |
| Kappa index ≥ 6.1 |  | >0.99 | 0.25 | 0.25 | 0.13 | **0.03↑** | **0.03↑** |
| Kappa index ≥ 7 |  |  | 0.25 | 0.25 | 0.13 | **0.03↑** | **0.03↑** |
| Kappa index ≥ 9 |  |  |  | >0.99 | >0.99 | 0.25 | 0.25 |
| Kappa index ≥ 11 |  |  |  |  | >0.99 | 0.25 | 0.25 |
| Kappa index ≥ 13 |  |  |  |  |  | 0.5 | 0.5 |
| Kappa index ≥ 15 |  |  |  |  |  |  | >0.99 |

Abbreviations: OCB, oligoclonal IgG bands. ↓: lower sensitivity; ↑: increased sensitivity or specificity.

**Supplementary table 4.** Sensitivity, specificity, positive predictive value and negative predictive value of kappa index, IgG index, Reiber for kappa free light chains and oligoclonal IgG bands and their combinations in optic nerve CIS patients.

| Test | Sensitivity (CI 95%) | Specificity (CI 95%) | PPV (CI 95%) | NPV (CI 95%) |
| --- | --- | --- | --- | --- |
| OCB | 98.2% (90.6% - 99.9%) | 95.0 (76.4% - 99.7%) | 98.2% (90.6% - 99.9%) | 95.0% (76.4% - 99.7%) |
| IgG index | 50.0% (37.3% - 62,7%) | 65.0% (43.3% - 81.9%) | 80.0% (64.1% - 90.0%) | 31.7% (19.6% - 47.0%) |
| Reiber´s KFLC | 100% (93.6% - 100%) | 70.0% (48.1% - 85.5%) | 90.3% (80.5% - 95.5%) | 100% (78.5% - 100%) |
| Kappa index ≥ 6.1 | 100% (93.6% - 100%) | 65.0% (43.3% - 81.9%) | 88.9% (78.8% - 94.5%) | 100% (77.2% - 100%) |
| Kappa index ≥ 7 | 100% (93.6% - 100%) | 65.0% (43.3% - 81.9%) | 88.9% (78.8% - 94.5%) | 100% (77.2% - 100%) |
| Kappa index ≥ 9 | 98.2% (90,6% - 99.9%) | 75.0% (53.1% - 88.8%) | 91.7% (81.9% - 96.4%) | 93.8% (71.7% - 99.7%) |
| Kappa index ≥ 11 | 85.7% (74.3% - 92.6%) | 75.0% (53.1% - 88.8%) | 90.6% (79.8% - 95.9%) | 65.2% (44.9% - 81.2%) |
| Kappa index ≥ 13 | 83.9% (72.2% - 91.3%) | 80.0% (58.4% - 91.9%) | 92.2% (81.5% - 96.9%) | 64.0% (44.5% - 79.8%) |
| Kappa index ≥ 15 | 83.9% (72.2% - 91.3%) | 85.0% (64.0% - 94.8%) | 94.0% (83.8% - 98.4%) | 65.4% (46.2% - 80.6%) |
| Kappa index ≥ 15 or OCB+ | 96.4% (87.7% - 99.4%) | 85.0% (64.0% - 94.8%) | 94.6% (85.4% - 98.5%) | 89.5% (68.6% - 98.1%) |

Abbreviations: CI; confidence interval; PPV, positive predictive value; NPV, negative predictive value; OCB, oligo-clonal bands; KFLC, kappa free light chains.

**Supplementary table 5.** Statistical differences in sensitivity and specificity in optic nerve CIS patients.

| **McNemar** | Kappa index ≥ 6.1 | Kappa index ≥ 7 | Kappa index ≥ 9 | Kappa index ≥ 11 | Kappa index ≥ 13 | Kappa index ≥ 15 | Kappa index ≥ 15 or OCB+ |
| --- | --- | --- | --- | --- | --- | --- | --- |
| **SENSITIVITY** | | | | | | | |
| Kappa index ≥ 6.1 |  | >0,99 | 0,32 | **0,005↓** | **0,003↓** | **0,003↓** | 0,16 |
| Kappa index ≥ 7 |  |  | 0,32 | **0,005↓** | **0,003↓** | **0,003↓** | 0,16 |
| Kappa index ≥ 9 |  |  |  | **0,008↓** | **0,005↓** | **0,005↓** | 0,56 |
| Kappa index ≥ 11 |  |  |  |  | 0,32 | 0,32 | **0,03↑** |
| Kappa index ≥ 13 |  |  |  |  |  | >0,99 | **0,008↑** |
| Kappa index ≥ 15 |  |  |  |  |  |  | **0,008↑** |
| **SPECIFICITY** | | | | | | | |
| Kappa index ≥ 6.1 |  | >0,99 | 0,16 | 0,16 | 0,08 | **0,03↑** | **0,03↑** |
| Kappa index ≥ 7 |  |  | 0,16 | 0,16 | 0,08 | **0,03↑** | **0,03↑** |
| Kappa index ≥ 9 |  |  |  | >0,99 | 0,32 | 0,08 | 0,08 |
| Kappa index ≥ 11 |  |  |  |  | 0,32 | 0,08 | 0,08 |
| Kappa index ≥ 13 |  |  |  |  |  | 0,16 | 0,16 |
| Kappa index ≥ 15 |  |  |  |  |  |  | >0,99 |

Abbreviations: OCB, oligoclonal IgG bands. ↓: lower sensitivity. ↑: increased sensitivity or specificity.

**Supplementary table 6.** Sensitivity, specificity, positive predictive value and negative predictive value of kappa index, IgG index and oligoclonal IgG bands and their combinations in any CIS patients except optic nerve CIS patients.

| Test | Sensitivity (CI 95%) | Specificity (CI 95%) | PPV (CI 95%) | NPV (CI 95%) |  |
| --- | --- | --- | --- | --- | --- |
| OCB | 96.0% (93.0% - 97.8%) | 88.2% (65.7% - 97.9%) | 99.3% (97.3% - 99.9%) | 57.7% (39.0% - 74.5%) | |
| IgG index | 75.1% (70.4% - 79.4%) | 76.9% (49.7% - 91.8%) | 98.9% (96.8% - 99.7%) | 10.2% (5.6% - 17.8%) | |
| Reiber´s KFLC | 99.3% (97.4% - 99.9%) | 88.2% (65.7% - 97.9%) | 99.3% (97.4% - 99.9%) | 88.2% (65.7% - 97.9%) | |
| Kappa index ≥ 6.1 | 92.1% (88.3% - 94.7%) | 88.2% (65.7% - 97.9%) | 99.2% (97.2% - 99.9%) | 40.5% (26.4% - 56.5%) | |
| Kappa index ≥ 7 | 91.3% (87.4% - 94.1%) | 88.2% (65.7% - 97.9%) | 99.2% (97.2% - 99.9%) | 38.5% (24.9% - 54.1%) | |
| Kappa index ≥ 9 | 88.5% (84.2% - 91.7% | 88.2% (65.7% - 97.9%) | 99.2% (97.1% - 99.9%) | 31.9% (20.4% - 46.2%) | |
| Kappa index ≥ 11 | 84.8% (80.1% - 88.6%) | 88.2% (65.7% - 97.9%) | 99.2% (97.0% - 99.9%) | 26.3% (16.7% - 39.0%) | |
| Kappa index ≥ 13 | 81.2% (76.2% - 85.4%) | 88.2% (65.7% - 97.9%) | 99.1% (96.9% - 99.8%) | 22.4% (14.1% - 33.7%) | |
| Kappa index ≥ 15 | 79.1% (73.9% - 83.4%) | 88.2% (65.7% - 97.9%) | 99.1% (96.8% - 99.8%) | 20.6% (12.9% - 31.2%) | |
| Kappa index ≥ 15 or OCB+ | 96.8% (93.9% - 98.3%) | 88.2% (65.7% - 97.9%) | 99.3% (97.3% - 99.9%) | 62.5% (42.7% - 78.8%) | |

Abbreviations: CI; confidence interval; PPV, positive predictive value; NPV, negative predictive value; OCB, oligo-clonal bands; KFLC, kappa free light chains.

**Supplementary table 7.** Statistical differences in sensitivity and specificity in all but optic nerve CIS patients

| **McNemar** | Kappa index ≥ 6.1 | Kappa index ≥ 7 | Kappa index ≥ 9 | Kappa index ≥ 11 | Kappa index ≥ 13 | Kappa index ≥ 15 | Kappa index ≥ 15 or OCB+ |
| --- | --- | --- | --- | --- | --- | --- | --- |
| **SENSITIVITY** | | | | | | | |
| Kappa index ≥ 6.1 |  | 0,16 | **0,002**↓ | **<0,001**↓ | **<0,001**↓ | **<0,001**↓ | **0,0005**↑ |
| Kappa index ≥ 7 |  |  | **0,005**↓ | **<0,001**↓ | **<0,001**↓ | **<0,001**↓ | **0,0002**↑ |
| Kappa index ≥ 9 |  |  |  | **0,002**↓ | **<0,001**↓ | **<0,001**↓ | **<0,001**↑ |
| Kappa index ≥ 11 |  |  |  |  | **0,0009**↓ | **0,0001**↓ | **<0,001**↑ |
| Kappa index ≥ 13 |  |  |  |  |  | **0,03**↓ | **<0,001**↑ |
| Kappa index ≥ 15 |  |  |  |  |  |  | **<0,001**↑ |
| **SPECIFICITY** | | | | | | | |
| Kappa index ≥ 6.1 |  | >0,99 | >0,99 | >0,99 | >0,99 | >0,99 | >0,99 |
| Kappa index ≥ 7 |  |  | >0,99 | >0,99 | >0,99 | >0,99 | >0,99 |
| Kappa index ≥ 9 |  |  |  | >0,99 | >0,99 | >0,99 | >0,99 |
| Kappa index ≥ 11 |  |  |  |  | >0,99 | >0,99 | >0,99 |
| Kappa index ≥ 13 |  |  |  |  |  | >0,99 | >0,99 |
| Kappa index ≥ 15 |  |  |  |  |  |  | >0,99 |

Abbreviations: OCB, oligoclonal IgG bands. ↓: lower sensitivity. ↑: increased sensitivity.

**Supplementary table 8.** Sensitivity and specificity of OCB and different kappa index cut-offs values for patients with brainstem clinical manifestations CIS.

| TEST | Sensitivity (CI 95%) | Specificity (CI 95%) | PPV (CI 95%) | NPV (CI 95%) |
| --- | --- | --- | --- | --- |
| OCB | 100% (95.0% - 100%) | 100% (51.3% -100%) | 100% (95.0% - 100%) | 100% (51.3% -100%) |
| IgG index | 71.2% (60.0% - 80.4%) | 98.1% (90.1% - 99.9%) | 80.0% (37.6% - 99.0%) | 16.0% (6.4% - 34.7%) |
| Reiber´s KFLC | 98.6% (92.6% - 99.9%) | 100% (51.3% -100%) | 100% (95.0% - 100%) | 50% (2.56% - 97.4%) |
| Kappa index ≥ 6.1 | 91.8% (83.2% - 96.2%) | 100% (51.3% -100%) | 100% (95.0% - 100%) | 14.3% (7,3% - 51.3%) |
| Kappa index ≥ 7 | 90.4% (81.5% - 95.3%) | 100% (51.3% -100%) | 100% (95.0% - 100%) | 12.5% (6.4% - 47.1%) |
| Kappa index ≥ 9 | 89.0% (79.8% - 94.3%) | 100% (51.3% -100%) | 100% (94.4% - 100%) | 11.1% (5.69% - 43.5%) |
| Kappa index ≥ 11 | 82.2% (71.9% - 89.3%) | 100% (51.3% -100%) | 100% (94.4% - 100%) | 7.0% (3.7% - 31.5%) |
| Kappa index ≥ 13 | 79.5% (68.8% - 87.1%) | 100% (51.3% -100%) | 100% (94.4% - 100%) | 6.0% (3.2% - 28.3%) |
| Kappa index ≥ 15 | 75.3% (64.4% - 83.8%) | 100% (51.3% -100%) | 100% (94.4% - 100%) | 5.3% (2.7% - 24.6%) |
| Kappa index ≥ 15 or OCB+ | 100% (95.0% - 100%) | 100% (51.3% -100%) | 100% (94.4% - 100%) | 100% (51.3% -100%) |

Abbreviations: CI; confidence interval; PPV, positive predictive value; NPV, negative predictive value; OCB, oligo-clonal bands; KFLC, kappa free light chains.

**Supplementary table 9.** Sensitivity and specificity of OCB and different kappa index cut-offs values for patients with spinal cord clinical manifestations CIS.

| TEST | Sensitivity (CI 95%) | Specificity (CI 95%) | PPV (CI 95%) | NPV (CI 95%) |
| --- | --- | --- | --- | --- |
| OCB | 95.8% (91.2% - 98.1%) | 90.0% (69.9% - 98.2%) | 98.6% (94.9% - 99.7%) | 75.0% (55.1% - 88.0%) |
| IgG index | 73.3% (66.7% - 79.1%) | 75.0% (53.1% - 88.8%) | 96.6% (92.3% - 98.6%) | 22.4% (14.1% - 33.7%) |
| Reiber´s KFLC | 100% (97.4% - 100%) | 90.0% (69.9% - 98.2%) | 98.6% (94.9% - 99.7%) | 100% (82.4% - 100%) |
| Kappa index ≥ 6.1 | 95.8% (91.2% - 98.1%) | 90.0% (69.9% - 98.2%) | 98.6% (94.9% - 99.7%) | 75.0% (55.1% - 88.0%) |
| Kappa index ≥ 7 | 95.1% (90.2% - 97.6%) | 90.0% (69.9% - 98.2%) | 98.6% (94.9% - 99.7%) | 72.0% (52.4% - 85.7%) |
| Kappa index ≥ 9 | 91.7% (86.0% - 95.2%) | 90.0% (69.9% - 98.2%) | 98.5% (94.7% - 99.7%) | 60.0% (42.3% - 75.4%) |
| Kappa index ≥ 11 | 90.3% (84.3% - 94.1%) | 90.0% (69.9% - 98.2%) | 98.5% (94.6% - 99.7%) | 56.3% (39.3% - 71.8%) |
| Kappa index ≥ 13 | 88.9% (82.7% - 93.0%) | 90.0% (69.9% - 98.2%) | 98.5% (94.6% - 99.7%) | 52.9% (36.7% - 68.6%) |
| Kappa index ≥ 15 | 88.2% (81.9% - 92.5%) | 90.0% (69.9% - 98.2%) | 98.5% (94.5% - 99.7%) | 51.4% (35.6% - 67.0%) |
| Kappa index ≥ 15 or OCB+ | 97.2% (93.1% - 98.9%) | 90.0% (69.9% - 98.2%) | 98.6% (95.0% - 99.8%) | 81.8% (61.5% - 92.7%) |

Abbreviations: CI; confidence interval; PPV, positive predictive value; NPV, negative predictive value; OCB, oligo-clonal bands; KFLC, kappa free light chains.

**Supplementary table 10.** Sensitivity and specificity of OCB and different kappa index cut-offs values for patients with cerebral hemisphere clinical manifestations CIS.

| TEST | Sensitivity (CI 95%) | Specificity (CI 95%) | PPV (CI 95%) | NPV (CI 95%) |
| --- | --- | --- | --- | --- |
| OCB | 84.4% (77.2% - 89.1%) | 100% (70.1% - 100%) | 100% (90.8% - 100%) | 56.3% (33.2% - 76.9%) |
| IgG index | 40.0% (27.0% - 54.6%) | 77.8% (45.3% - 96.1%) | 90.0% (69.9% - 98.2%) | 20.6% (10.4% - 36.8%) |
| Reiber´s KFLC | 95.5% (84.9% - 99.2%) | 100% (70.1% - 100%) | 100% (90.8% - 100%) | 66.7% (37.2% - 80.1%) |
| Kappa index ≥ 6.1 | 75.6% (68.1% - 82.0%) | 100% (70.1% - 100%) | 100% (89.9% - 100%) | 45.0% (25.8% - 65.8%) |
| Kappa index ≥ 7 | 75.6% (68.1% - 82.0%) | 100% (70.1% - 100%) | 100% (89.9% - 100%) | 45.0% (25.8% - 65.8%) |
| Kappa index ≥ 9 | 71.1% (62.2% - 77.0%) | 100% (70.1% - 100%) | 100% (89.9% - 100%) | 40.9% (23.3% - 61.3%) |
| Kappa index ≥ 11 | 64.4% (56.4% - 71.9%) | 100% (70.1% - 100%) | 100% (88.3% - 100%) | 36.0% (20.3% - 55.5%) |
| Kappa index ≥ 13 | 48.9% (40.6% - 56.7%) | 100% (70.1% - 100%) | 100% (85.1% - 100%) | 28.1% (15.6% - 45.4%) |
| Kappa index ≥ 15 | 44.4% (36.6% - 52.6%) | 100% (70.1% - 100%) | 100 (83.9% - 100%) | 26.5% (14.6% - 43.1%) |
| Kappa index ≥ 15 or OCB+ | 84.4% (77.2% - 89.1%) | 100% (70.1% - 100%) | 100% (90.8% - 100%) | 56.3% (33.2% - 76.9%) |

Abbreviations: CI; confidence interval; PPV, positive predictive value; NPV, negative predictive value; OCB, oligo-clonal bands; KFLC, kappa free light chains.

**Supplementary table 11.** Sensitivity and specificity of OCB and different kappa index cut-offs values for patients with multifocal clinical manifestations CIS.

| TEST | Sensitivity (CI 95%) | Specificity (CI 95%) | PPV (CI 95%) | NPV (CI 95%) |
| --- | --- | --- | --- | --- |
| OCB | 100% (67.6% - 100%) | 100% (17.8% - 100%) | 100% (67.6% - 100%) | 100% (17.8% - 100%) |
| Reiber´s KFLC | 100% (67.6% - 100%) | 100% (17.8% - 100%) | 100% (67.6% - 100%) | 100% (17.8% - 100%) |
| IgG index | 87.5% (52.9% - 99.4%) | 100% (17.8% - 100%) | 100% (61.0% - 100%) | 66.7% (11.9% - 98.3%) |
| Kappa index ≥ 6.1 | 87.5% (52.9% - 99.4%) | 100% (17.8% - 100%) | 100% (64.6% - 100%) | 66.7% (11.9% - 98.3%) |
| Kappa index ≥ 7 | 87.5% (52.9% - 99.4%) | 100% (17.8% - 100%) | 100% (64.6% - 100%) | 66.7% (11.9% - 98.3%) |
| Kappa index ≥ 9 | 87.5% (52.9% - 99.4%) | 100% (17.8% - 100%) | 100% (64.6% - 100%) | 66.7% (11.9% - 98.3%) |
| Kappa index ≥ 11 | 87.5% (52.9% - 99.4%) | 100% (17.8% - 100%) | 100% (64.6% - 100%) | 66.7% (11.9% - 98.3%) |
| Kappa index ≥ 13 | 87.5% (52.9% - 99.4%) | 100% (17.8% - 100%) | 100% (64.6% - 100%) | 66.7% (11.9% - 98.3%) |
| Kappa index ≥ 15 | 87.5% (52.9% - 99.4%) | 100% (17.8% - 100%) | 100% (64.6% - 100%) | 66.7% (11.9% - 98.3%) |
| Kappa index ≥ 15 or OCB+ | 100% (67.6% - 100%) | 100% (17.8% - 100%) | 100% (67.6% - 100%) | 100% (17.8% - 100%) |

Abbreviations: CI; confidence interval; PPV, positive predictive value; NPV, negative predictive value; OCB, oligo-clonal bands; KFLC, kappa free light chains.
